# Association and clinical utility of *NAT2* in the prediction of isoniazid-induced liver injury in Singaporean patients

**DOI:** 10.1371/journal.pone.0186200

**Published:** 2017-10-16

**Authors:** Sze Ling Chan, Angeline Poh Gek Chua, Folefac Aminkeng, Cynthia Bin Eng Chee, Shengnan Jin, Marie Loh, Suay Hong Gan, Yee Tang Wang, Liam R. Brunham

**Affiliations:** 1 Translational Laboratory in Genetic Medicine, Agency for Science, Technology and Research, Singapore and the National University of Singapore, Singapore; 2 Department of Respiratory Medicine, Tan Tock Seng Hospital, Singapore; 3 School of Laboratory Medicine and Life Science, Wenzhou Medical University, Wenzhou, China; 4 Department of Medicine, Centre for Heart Lung Innovation, University of British Columbia, Vancouver, Canada; Rutgers Biomedical and Health Sciences, UNITED STATES

## Abstract

**Background and aims:**

Isoniazid (INH) is part of the first-line-therapy for tuberculosis (TB) but can cause drug-induced liver injury (DILI). Several candidate single nucleotide polymorphisms (SNPs) have been previously identified but the clinical utility of these SNPs in the prediction of INH-DILI remains uncertain. The aim of this study was to assess the association between selected candidate SNPs and the risk of INH-DILI and to assess the clinical validity of associated variants in a Singaporean population.

**Methods:**

This was a case-control study where 24 INH-DILI cases and 79 controls were recruited from the TB control unit in a tertiary hospital. Logistic regression was used to test for the association between candidate SNPs and INH-DILI. NAT2 acetylator status was inferred from genotypes and tested for association with INH-DILI. Finally, clinical validity measures were estimated for significant variants.

**Results:**

Two SNPs in *NAT2* (rs1041983 and rs1495741) and NAT2 slow acetylators (SA) were significantly associated with INH-DILI (OR (95% CI) = 13.86 (4.30–44.70), 0.10 (0.03–0.33) and 9.98 (3.32–33.80), respectively). Based on an INH-DILI prevalence of 10%, the sensitivity, specificity, positive and negative predictive values of NAT2 SA were 75%, 78%, 28% and 97%, respectively. The population attributable fraction (PAF) and number needed to test (NNT) for NAT2 SA were estimated to be 0.67 and 4.08, respectively. A model with clinical and NAT2 acetylator status provided significantly better prediction for INH-DILI than a clinical model alone (area under receiver operating characteristic curve = 0.863 vs. 0.766, respectively, p = 0.027).

**Conclusions:**

We show the association between NAT2 SA and INH-DILI in a Singaporean population and demonstrated its clinical utility in the prediction of INH-DILI.

## Introduction

Tuberculosis (TB) is one of the top 10 causes of death world-wide, with an estimated 10.4 million new cases and 1.8 million TB-related deaths worldwide in 2015 [1]. Isoniazid (INH) is an effective anti-mycobacterium agent and is part of first-line 4-drug therapy for TB that also includes rifampicin, pyrazinamide and ethambutol [1]. However, INH is associated with drug-induced liver injury (DILI) that can range from asymptomatic elevations in liver enzymes in 10–20% of patients, to clinically significant hepatitis and acute liver failure in 0.5–1% of patients [2]. Risk factors for INH-DILI include age, female gender, black race, alcoholism, pre-existing liver disease, and concomitant medications such as rifampicin and pyrazinamide [2].

The molecular mechanisms of anti-TB DILI are not fully understood, but it is thought accumulation of toxic intermediate metabolites plays an important role [3]. Pharmacogenomic studies have been mainly focused on candidate genes involved in INH biotransformation and metabolism, including N-acetyltransferase 2 (*NAT2*), cytochrome P450 2E1 (*CYP2E1*) and glutathione-S-transferase genes (*GSTT1*, *GSTM1* and *GSTP1*). In addition, INH and some of its metabolites are readily oxidized to reactive species, which may contribute to liver damage [4]. INH-DILI typically occurs within 2 weeks to 6 months after commencing therapy [2]. The delayed nature of INH-DILI also points to a possible immune component [4]. As such, genes in the antioxidant and detoxification pathways [5–8], human-leukocyte antigen system [9,10] and tumor necrosis factor-α [11] have also been studied.

Meta-analyses have consistently shown that NAT2 slow acetylators (SA) have a higher risk of INH-DILI, with an odds ratio (OR) of around 3.1 [12,13]. Seven common coding single nucleotide polymorphisms (SNPs) (rs1801279, rs1041983, rs1801280, rs1799929, rs1799930, rs1208 and rs1799931) define 34 haplotypes, but most studies only genotype a subset to infer acetylator status [14]. Recently, a tag SNP, rs1495741, located 14.5kb 3’ of *NAT2* has been associated with INH-DILI, although it may not be a good surrogate for acetylator status [14,15].

In *CYP2E1*, two polymorphisms are most commonly studied (*RsaI/PstI* (rs2031920/rs3813867) and *DraI* (rs6413432)). rs2031920 and rs3813867 are in complete linkage disequilibrium in East Asians [16]. A recent meta-analysis found that the *RsaI/PstI* polymorphism *c1/c1* (major allele for both SNPs) confers a higher risk of INH-DILI with an OR of 1.32 compared to the c1/c2 or c2/c2 genotypes, but no increase in risk for the *DraI* polymorphism [16].

These candidate SNPs have been studied in diverse populations including several of Asian descent (Chinese, Xinjiang Uyghurs, Japanese, Korean, Indian, Taiwanese and Indonesian) [15,17–39]. However, no previous study has been conducted in a Singaporean population. The 3 major ethnic groups in Singapore, Chinese, Malays and Indians, originated largely from Southern China, Peninsula Malaysia and Indonesia, and Southern India, respectively [40]. Results derived from the Singaporean population may therefore have broad applicability to populations that have not been well represented in previous candidate gene studies. Malays in particular, have not been included in INH-DILI studies apart from one focused on *NAT2* [35]. Representation of these populations is crucial since India, China and Indonesia are among the countries with the greatest burden of TB burden worldwide [1].

To facilitate the clinical implementation of prospective pharmacogenomic screening for INH-DILI in these South and Southeast Asian countries, it is imperative to determine the genetic and clinical relevance of INH-DILI pharmacogenomic biomarkers in representative populations. Therefore, the objective of this study was to assess the association between selected candidate SNPs and the risk of INH-DILI, and to assess the clinical validity of associated variants in a multiethnic Singaporean population representative of the Southeast Asian region.

## Results

### Clinical and demographic characteristics

A total of 104 patients were recruited (S1 Fig). One patient failed genotyping quality control (QC) due to sex mismatch, leaving 103 patients (24 cases and 79 controls) for analysis. No samples were removed due to high identity-by-state (IBS). The clinical characteristics of cases and controls are shown in Table 1. Most cases had grade 2 (41.7%) or grade 3 (54.2%) INH-DILI and only 1 patient had grade 4 INH-DILI. The median onset time for INH-DILI was 17.5 days (range 4–111 days), which is consistent with the typical onset time of 2 weeks to 6 months [2]. Cases were more likely to be female than controls (p = 1.80 x 10^−3^) and were more likely to be of ‘Other’ ethnicity (Table 1).

**Table 1:** Demographic and clinical characteristics of Singaporean patients diagnosed with TB and treated with INH.

Clinical and Demographic Characteristics	All Patients (n = 103)	INH-DILI Cases (n = 24)	Controls (n = 79)	P value*
Age, mean (sd)	51.1 (14.5)	51.6 (16.8)	50.9 (13.9)	0.728^‡^
Male gender, n (%)	68 (66.0)	9 (37.5)	59 (74.7)	1.80 x 10^−3^^†^
Ethnicity, n (%)				0.034^†^
Chinese	69 (67.0)	12 (50.0)	57 (72.2)	0.051^¶^
Malays	15 (14.6)	5 (20.8)	10 (12.7)	0.333^¶^
Indians	8 (7.8)	1 (4.2)	7 (8.9)	0.677^¶^
Others	11 (10.7)	6 (25.0)	5 (6.3)	0.018^¶^
BMI, mean (sd)	21.5 (3.7)	21.5 (3.8)	21.4 (3.7)	0.949^§^
TB type, n(%)				0.934^†^
Pulmonary	73 (70.9)	17 (70.8)	56 (70.9)	
Extrapulmonary	19 (18.4)	4 (16.7)	15 (19.0)	
Both	11 (10.7)	3 (12.5)	8 (10.1)	
Drug doses (mg), median (range)				
Isoniazid	300 (200–386^₤^)	300 (200–300)	300 (200–386^₤^)	0.648^‡^
Rifampicin	600 (257^₤^–600)	600 (300–600)	600 (257^₤^–600)	0.149^‡^
Pyrazinamide	1250 (643^₤^–2000)	1250 (1000–1750)	1250 (643^₤^–2000)	0.590^‡^
Ethambutol	1000 (400–1600)	950 (600–1400)	1000 (400–1600)	0.204^‡^
Received hepatotoxic concomitant medications^, n(%)	22 (21.3)	5 (20.8)	17 (21.5)	1^†^
NIH Grade, n (%)				
0		0	79 (100)	-
2		10 (41.7)	0	-
3		13 (54.2)	0	-
4		1 (4.2)	0	-
Baseline LFT, median (range)				
AST (U/L)		22 (14–48)	21 (11–69)	-
ALT (U/L)		18 (8–46)	18 (6–64)	-
Onset or follow up LFT, median (range)				
AST (U/L)		212 (104–1401)	22 (9–42)	-
ALT (U/L)		199.5 (58–684)	17 (5–42)	-

*P* < 0.05 was considered statistically significant and was used for the selection of covariates for the subsequent genomic association analysis

*For association between clinical characteristic and case-control status,

^‡^Mann-Whitney U test,

^†^Fisher’s exact test,

^¶^each category compared against all others combined,

^§^t-test,

^₤^one patient on renal dialysis received thrice weekly doses of all drugs.

^Hepatotoxic medications considered here include atorvastatin, simvastatin, ibuprofen, clopidogrel, fenofibrate, paracetamol, acarbose, amitriptyline and tolbutamide. These medications were among drugs with likelihood categories A and B in NIH livertox database.

ALT: alanine transaminase, AST: aspartate transaminase, BMI: body mass index, INH-DILI: isoniazid-induced liver injury, LFT: liver function test, NIH: National Institute of Health, mg: milligram, sd: standard deviation, TB: tuberculosis

After removing SNPs with call rate <95% or deviation from Hardy-Weinberg Equilibrium (HWE) within Chinese patients, 536859 SNPs remained. Principal components analysis (PCA) performed using our genome-wide SNP data showed distinct clustering according to self-reported ethnicity, except for ‘Others’ which was spread between the Chinese and Malay clusters (S2 Fig). Although Mann-Whitney U tests on the first 10 principal components (PCs) by case control status were not statistically significant, we used the first 2 PCs to adjust for ethnic differences in subsequent analyses based on the fact that there were different ethnicities in our cohort, scree plot of the eigenvalues (S3 Fig) and visual inspection of PCA plot (S2 Fig).

### Pharmacogenomic association analysis

We performed a comprehensive literature review of INH-DILI and identified 20 candidate SNPs with prior evidence of association with INH-DILI, minor allele frequency (MAF) > 0.05 in at least 1 population and present in our genotyping panel (S1 Table), which we tested for association in our cohort. Of these 20 candidate SNPs, 2 SNPs (rs1041983 and rs1495741), both in the *NAT2* gene, were significantly associated with INH-DILI in our cohort after correction for multiple correlated tests. The recessive model was most significant for rs1041983 (OR (95% confidence interval (CI)) = 13.86 (4.30–44.70), adj P = 4.754 x 10^−4^), whereas the dominant model was most significant for rs1495741 (OR (95% CI) = 0.10 (0.03–0.33), adj P = 0.004) (Table 2 & S2 Table). Analyses using the first 5 PCs, 20 PCs or self-reported ethnicity instead of the first 2 PCs yielded very similar results. Association with INH-DILI severity by ordinal logistic regression also yielded very similar results (S3 Table). Cluster plots of these 2 SNPs showed clear clustering of genotypes (S4 Fig).

**Table 2:** Pharmacogenomic association of *NAT2* variants in the Singaporean population.

Gene	SNP	Maj/ min	MAF		Additive			Dominant			Recessive		
			Cases	Ctrl	OR (95% CI)	P	Adj P	OR (95% CI)	P	Adj P	OR (95% CI)	P	Adj P
*NAT2*	rs1041983	G/A	0.792	0.367	**6.34 (2.54–15.82)**	**7.667 x 10**^**−5**^	**0.003**	5.72 (1.18–27.69)	0.030	0.597	**13.86 (4.30–44.70)**	**1.078 x 10**^**−5**^	**4.754 x 10**^**−4**^
*NAT2*	rs1495741	A/G	0.167	0.551	**0.21 (0.09–0.52)**	**6.267 x 10**^**−4**^	**0.024**	**0.10 (0.03–0.33)**	**1.084 x 10**^**−4**^	**0.004**	0.25 (0.05–1.21)	0.085	0.901
*NAT2*	rs1799929	G/A	0.042	0.076	0.36 (0.07–1.95)	0.235	0.995	0.36 (0.07–1.95)	0.235	0.995	NA*		
*NAT2*	rs1799930	G/A	0.5	0.222	2.85 (1.32–6.16)	0.008	0.226	2.16 (0.74–6.28)	0.159	0.980	12.00 (2.49–57.84)	0.002	0.069
*NAT2*	rs1799931	G/A	0.292	0.139	2.85 (1.20–6.77)	0.017	0.420	3.10 (1.05–9.15)	0.040	0.691	7.33 (0.96–56.24)	0.055	0.790
*NAT2*	rs1801280	A/G	0.042	0.095	0.30 (0.05–1.62)	0.161	0.978	0.30 (0.05–1.68)	0.169	0.982	NA*		

This table shows the association results from logistic regression with gender, PC1 and PC2 as covariates. P values were adjusted for 55 multiple correlated tests using the p_ACT procedure. Significant SNPs (Adj P <0.05) are bolded. Only results for *NAT2* are shown here.

*Logistic regression could not be performed due to absence of patients with homozygous variant in either cases or controls.

Adj P: adjusted P value, CI: confidence interval, Ctrl: controls, MAF: minor allele frequency, Maj: major allele, Min: minor allele, OR: odds ratio

**Fig 1:**
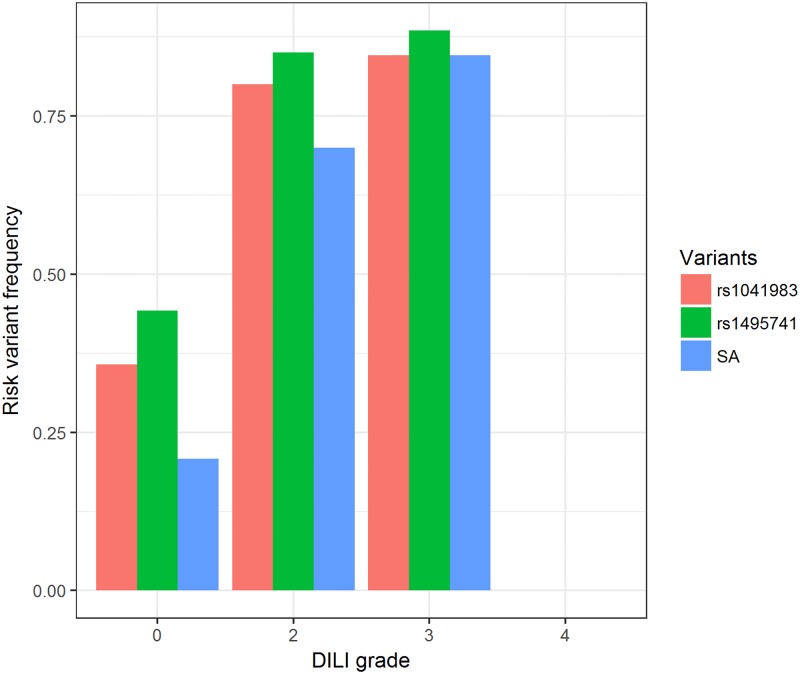
Risk allele frequency of associated *NAT2* variants by DILI grade. The frequencies of the risk variants (rs1041983 A, rs1495741 A and NAT2 SA) increase with DILI grade. There was only 1 patient with grade 4 DILI, who was homozygous for the non-risk variants. DILI: drug-induced liver injury, SA: slow acetylators.

We next inferred acetylator phenotype using a 4 SNP panel, which did not include either of the 2 significant SNPs [14]. Thirty-five patients (34%) were predicted to be SA. SA status was significantly associated with INH-DILI compared to intermediate acetylator (IA) + rapid acetylator (RA) (OR (95% CI) = 9.98 (3.32–33.80), logistic regression p = 8.36 x 10^−5^). Results from Fisher’s exact test were similar (S4 Table). The frequencies of the risk alleles showed an increasing trend with DILI grade (Fig 1).

Genetic associations may be specific to certain populations. To determine if the associations we observed were consistent across all ethnic groups, we also explored the association between NAT2 acetylator status and the 2 significant SNPs within the 3 major ethnic groups in our cohort. The effect sizes remained similar in Chinese but were smaller and consistent in direction in Malays and Indians (S5 Table).

### Pharmacogenomic risk prediction analyses

**Fig 2:**
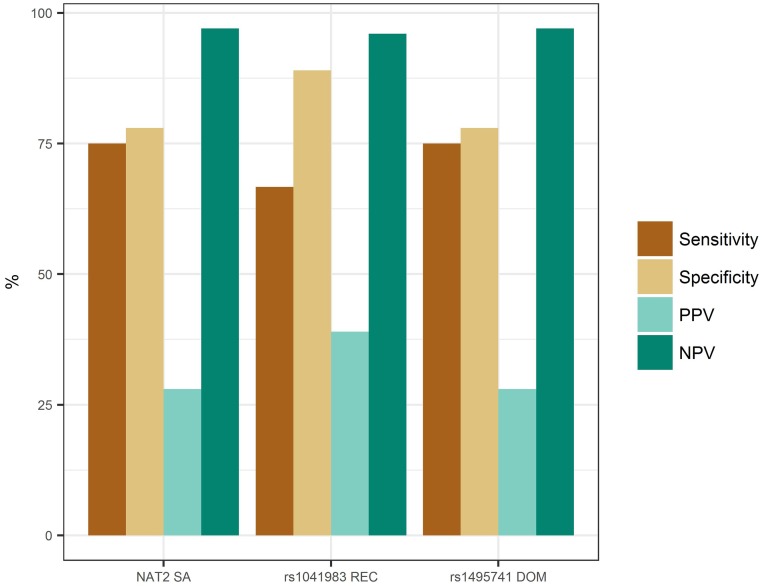
Clinical validity of NAT2 acetylator status and significant SNPs. Prevalence of 10% is assumed and the risk genotypes for all variants were used for the calculation of clinical validity measures. For both rs1041983 and rs1495741, the homozygous AA genotype is the risk genotype since GA/GG is protective. DOM: dominant, NPV: negative predictive value, PPV: positive predictive value, REC: recessive, SA: slow acetylators.

Next, we investigated the accuracy and clinical significance of NAT2 acetylator status and the 2 significant *NAT2* SNPs in predicting INH-DILI. Based on the estimated prevalence of serum aminotransferase elevations of 10% [2], we estimated the sensitivity, specificity, positive predictive value (PPV) and negative predictive value (NPV) of predicting INH-DILI at a prevalence of 10% from NAT2 SA status to be 75%, 78%, 28% and 97%, respectively. Interestingly, the 2 significant *NAT2* SNPs also demonstrated similar performance measures, though rs1041983 had slightly lower sensitivity but higher specificity and PPV (Fig 2). As PPV and NPV are dependent on prevalence of the outcome, we also calculated these values for a range of prevalence from 5–20%. While PPV varied considerably (16–47%), NPV was within a narrow range of 93–98% (S6 Table). The population attributable fraction (PAF) and number needed to test (NNT) for NAT2 SA were estimated to be 0.67 and 4.08, respectively.

**Fig 3:**
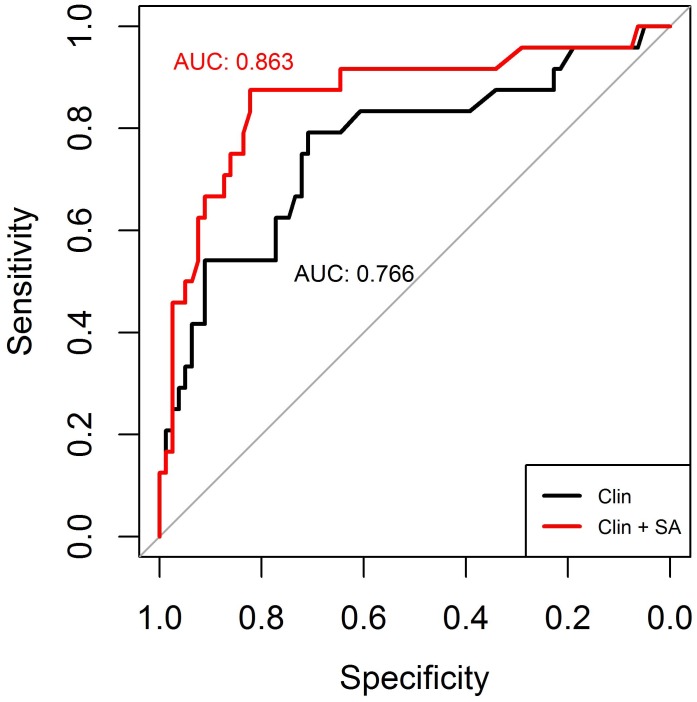
Predictive value of NAT2 acetylator status. Predictive values were evaluated using receiver operating characteristic (ROC) curves and expressed as area-under-curve (AUC), which is a summary measure of the sensitivity and specificity. The clinical model (clin) consists of age, gender and self-reported ethnicity. SA: slow acetylators.

To examine if *NAT2* significantly improves INH-DILI prediction over clinical factors, we then compared the predictive accuracy of a model using clinical variables alone to models that included NAT2 acetylator status using receiver operating characteristic (ROC) curves (Fig 3) and Youden’s index. The addition of NAT2 acetylator status provided significantly better prediction over a model with clinical variables alone (area under the curve (AUC) = 0.863 vs. 0.766, respectively, p = 0.027) (Fig 3). We also assessed the additional contributions of rs1041983 and rs1495741 individually as a comparison to NAT2 acetylator status. Models with clinical factors and the 2 SNPs separately also performed similarly, with AUCs of 0.861 (rs1495741) and 0.853 (rs1041983) (S5 Fig). Interestingly, both models with clinical factors and rs1495741 or rs1041983 were significantly better than clinical factors alone (p = 0.029 and 0.038, respectively), but not significantly different from one with clinical factors and NAT2 acetylator status (p = 0.678 and 0.592, respectively). The Youden’s indexes for clinical factors alone, clinical and NAT2 acetylator status, rs1495741 or rs1041983 were 0.500, 0.698, 0.672 and 0.656, respectively.

We further explored the correlation between the 2 SNPs and NAT2 acetylator status in order to determine how well these SNPs tag NAT2 acetylator status in the Singaporean populations. We found that the rs1495741 AA genotype had almost complete concordance (98.1%) with the SA phenotype (S7 Table).

## Discussion

Here we assessed the association of previously identified pharmacogenomic biomarkers for INH-DILI in a multiethnic Singaporean population. The major findings of this study are that we confirmed the association for 2 *NAT2* variants and NAT2 SA status with INH-DILI in this population for the first time. The effect size of 9.98 (95% CI: 3.32–33.8) that we observed was larger than the pooled ORs of 3.1 (3.10 (95% CI: 2.47–3.88) and 3.08 (95% CI: 2.29–4.15)) in prior meta-analyses, although the 95% CIs overlap [12,13]. The effect size we found was also similar to that reported in some studies in Japanese and Iranian populations [17,22,41]. This is the first study to report on the association of NAT2 variants and INH-DILI in a Singaporean population, and adds to the body of literature on the importance of this marker as a predictor of INH-DILI. We also demonstrate the potential clinical utility of NAT2 genotyping for INH-DILI prediction in these Southeast Asian populations.

Our study had >75% power to detect associations for variants with MAF ≥ 0.15 and allelic OR ≥ 4.5 at a significance level of 0.003 (Bonferroni corrected alpha level for 17 independent SNPs). The MAF of majority of the candidate SNPs outside *NAT2* (except *TNF* rs1800629) were > 0.15, so the lack of replication of these variants in our study was likely because the true effect sizes were too small to be detected.

We further demonstrated the clinical validity of testing for *NAT2* variants for the prediction of INH-DILI in patients receiving anti-TB drugs. Based on the effect size in our study, *NAT2* is an effective test for ruling out INH-DILI if patients are found to not have SA status, owing to the high NPV. Importantly, the NPV is robust to uncertainties in the INH-DILI prevalence. This is potentially clinical significant, as it suggests that patients found to be non-SA could safely undergo treatment with INH with limited monitoring for DILI and high confidence that INH-DILI would not occur. Conversely, patients with SA status would likely benefit from closer surveillance for the development of DILI. Two-thirds of INH-DILI cases can be attributed to the presence of NAT2 SA, and about 4 patients would need to be tested to prevent one case, if followed by an intervention that effectively mitigates the risk of INH-DILI. *NAT2* was also shown to significantly improve INH-DILI prediction over clinical factors alone. To our knowledge, this is the first study to include an assessment of the clinical validity of *NAT2* for INH-DILI, which is a necessary step towards clinical implementation of this drug-gene pair. Future studies will be able to formally assess the cost-effectiveness of NAT2 genotyping based on the clinical validity of the test as determined in our study.

Our results add to the body of evidence supporting the use of NAT2 acetylator status for prediction of INH-DILI. For individuals found to be at high genetic risk of INH-DILI, dose-adjustment based on *NAT2* genotype may represent a strategy to reduce the risk of DILI. A clinical trial of pharmacogenomic-guided INH dosing in a Japanese population demonstrated a significant reduction in INH-DILI when NAT2 SA were administered a reduced, without compromising treatment effectiveness [42]. Another pilot study linking *NAT2* genotype and INH serum concentration also supports pharmacogenetic-guided INH dosage regimens [43]. Further work is needed to establish evidence-based genotype specific clinical recommendations for NAT2 [44].

While NAT2 acetylator status has been consistently associated with INH-DILI, results for individual *NAT2* SNPs are much more variable. For example, rs1799930 was significantly associated with INH-DILI in some studies [23,27,32,34,35,45,46] but not others [24,29,31,47,48]. One possibility for this observation could be the imperfect tagging of NAT2 acetylator status by a single SNP, which we also observed in our study. A tag SNP for NAT2 acetylator status (rs1495741) was identified in a bladder cancer GWAS in Europeans [49]. Other studies have also found high concordance between rs1495741 and NAT2 acetylator status in Asians, Europeans and admixed Americans, but not in Africans [15,50]. Consistent with these observations, we also found high concordance between rs1495741 and NAT2 acetylator status in our cohort. The presence of African Americans in the study of Hein *et al*. may explain the high misclassification rate for rs1495741 reported in that study [14]. Taken together, our results suggest rs1495741 could be used to tag NAT2 SA with high accuracy in Southeast Asian populations.

There are several limitations to our study. Firstly, we did not have sufficient Malay and Indian patients to thoroughly explore the ethnic specificity of the associations in those sub-groups. However, it appears that the effect is consistent across the 3 major ethnic groups though the associations in Malay and Indian populations alone did not reach statistical significance due to small sample size. Secondly, some variants previously associated with INH-DILI were not included due to the nature of our genotyping assay. However, a review of these excluded variants found none of sufficient priority in terms of strength of evidence. Most variants were from single studies or were not significant in meta-analyses (*GSTM1* and *CYP2E1* rs6413432). We therefore chose not to genotype or impute them in our study. Thirdly, our study focused on specific candidate gene variants, and did not identify new genetic variants for INH-DILI. Future studies which combine multiple cohorts will be needed to perform GWAS for INH-DILI with appropriately powered sample sizes. Lastly, it was not possible to identify definitively the causative drug for the DILI as patients were taking 4 drugs simultaneously, but INH has the highest hepatotoxic potential among the drugs. All DILI cases were re-challenged and 80% of them tolerated INH upon re-challenge and completed their treatment. However, this is in line with reports that up to 80% of suspected INH-DILI cases tolerate reintroduction of treatment after the resolution of the initial injury [2].

In conclusion, we have reestablished the association between NAT2 SA and INH-DILI in a Singaporean population and demonstrated its clinical validity in prediction of INH-DILI. Clinical studies and pharmacoeconomic analyses are now needed to support the clinical implementation of *NAT2* pharmacogenetic testing for INH-DILI in Singapore.

## Material and methods

### Recruitment and detailed clinical characterization of patients

Cases of INH-DILI were defined as patients with Common Terminology Criteria for Adverse Events (CTCAE) v4.03 grade 2 and above for levels of aspartate transaminase (AST) and/or alanine transaminase (ALT) (i.e. >2.5x upper limit of normal (ULN)) [51], and were recruited retrospectively. Patients with active TB from 2014 to January 2016 were identified from the National TB registry, and those treated at the TB Control Unit (TBCU) in Tan Tock Seng Hospital were screened. Potential cases were shortlisted if their TB treatment was interrupted because of drug-induced liver reactions. A trained research nurse reviewed the medical records of these patients to determine if they met the inclusion criteria: i) undergoing treatment of active TB, ii) on anti-TB drugs which should include INH, rifampicin, pyrazinamide, and ethambutol, and iii) 21 to 95 years old. Patients were excluded if they had any of the following: i) unable to provide informed consent, ii) pregnant or breast feeding, iii) alcoholism, iv) chronic hepatitis B or C and other types of hepatitis or v) HIV co-infection. There was no limitation on the co-administration of drugs unrelated to TB treatment but we also assessed the possible contribution of potentially hepatotoxic drugs. For this purpose, drugs classified in categories A (>50 published reports) and B (13–50 published reports) for likelihood of causing DILI were considered hepatotoxic [52,53]. Eligible cases were then recalled for informed consent and blood sampling.

Control patients were recruited from October 2015 to April 2016 and were eligible if they had completed 6 months of TB treatment in TBCU with no symptoms of hepatotoxicity during treatment, and had normal post-treatment liver function tests (LFTs). All patients received their anti-TB treatment under direct observed therapy and all controls were adherent to therapy.

Blood or saliva was collected from recruited patients and deoxyribonucleic acid (DNA) extracted using the prepIT-L2P kit (Genotek, Ottawa, Canada) and DNeasy Blood & Tissue Kit (Qiagen GmbH, Hilden, Germany), respectively, according to manufacturer’s instructions. Clinical data were collected from all patients, including demographics, drug treatment details, baseline and follow-up LFTs, which occurred at the point of onset for cases or 6 months after treatment initiation for controls. All patients gave their written informed consent and the study was approved by the National Healthcare Group Domain Specific Review Board.

### Genotyping and quality control

All samples were genotyped using the Illumina HumanOmniExpress-24 beadchip v1.2 (Illumina, San Diego, CA). To verify the accuracy of all genotyping results, multiple positive and negative (water) controls as well as duplicate samples were included in the assays. No genotyping errors were detected with the use of these controls. To remove any samples or SNPs with genotyping failure or errors, QC filtering was performed to remove samples with high IBS, discordant sex, excess heterozygosity or call rate <95%, and SNPs with call rate <95%, deviation from HWE in Chinese (p <1.0 x 10^−6^). All Chinese patients (cases and controls) were chosen as the population for HWE testing to remove SNPs with genotyping errors in view of the presence of population mixture and small sample size. PCA was performed and we included PCs as covariates to adjust for population structure. Cluster plots of SNPs reaching statistical significance were inspected visually.

### Candidate SNP selection

A comprehensive literature search was performed via PubMed to identify previous pharmacogenomic studies on INH-DILI using the key words (isoniazid OR tuberculosis) AND (liver injury OR hepatitis OR hepatotoxicity) AND (pharmacogen* OR gene OR genetic OR allele* OR variant*) on 7 Oct 2016. We also searched the publication list for INH in the Pharmacogenomics Knowledgebase (PharmGKB) [54], a publicly available resource with manually curated knowledge on the impact of human genetic variation on drug response, to supplement the primary literature search. Genetic variants that have been found to be associated with INH-DILI at least once were then screened for their allele frequencies in Chinese, Malays and Indians using the Singapore Genome Variation Project database [40] or 1000 genomes (Southern Han Chinese (CHS) and Sri Lankan Tamil from the UK (STU), which has previously been shown to be very similar to Singaporean Indians) [55,56], and their presence in the genotyping panel.

### Statistical analysis

The primary study was designed as a case control study comparing a group of adult TB patients with INH-DILI with a drug matched control group without INH-DILI. We compared clinical characteristics between cases and controls to identify any that were significantly different (P < 0.05) between the 2 groups using t-test, Mann-Whitney U test, chi-square test or Fisher’s exact test as appropriate. The associations between the candidate SNPs and INH-DILI were tested using logistic regression under different genetic models (additive/dominant/recessive), with gender and the first 2 PCs as covariates. Results were presented as P-values and OR with 95% CIs. To adjust for multiple testing, we calculated adjusted P-values using P_ACT_, a computationally efficient method which takes into account correlation due to linkage disequilibrium and different genetic models [57]. Adjusted P-values of <0.05 were considered statistically significant and the genetic model with the lowest P-value was used for further analyses. The distribution of the individual alleles and genotypes between cases and controls for the associated variants were also assessed and the P-values and ORs with 95% CIs for each of the respective alleles and genotypes estimated using Fisher’s exact test.

We inferred NAT2 acetylator status using a 4 SNP panel (rs1801280, rs1799930, rs1799931 and rs1801279), where RA were homozygous common for all SNPs, IA were heterozygous for any SNP and SA were heterozygous for ≥2 SNPs or homozygous variant for at least 1 SNP [14]. rs1801279 was not included in our candidate SNP list as it was not present on our genotyping panel and expected to be monomorphic in Chinese, Malays and Indians (S1 Table). We therefore considered this SNP to be homozygous wild type in our cohort. The association between NAT2 acetylator status and INH-DILI was tested using logistic regression with covariates included, as well as Fisher’s exact test. All statistical tests performed were 2-sided.

To evaluate the clinical utility of NAT2 acetylator status and associated SNPs, we calculated the sensitivity, specificity, and estimated the PPV and NPV using Baye’s theorem assuming a prevalence of 10% [58]. To take into account uncertainty in the estimation of INH-DILI prevalence, we also performed a sensitivity analysis for the PPV and NPV over a range of prevalences (5–20%). We further calculated the PAF and NNT to detect 1 case of INH-DILI according to the following formulas,

$$PAF=\frac{IP_{t}-IP_{o}}{IP_{t}}$$ (1)

$$NNT=\frac{1}{ARR}$$ (2)

where *IP*_*t*_ = incidence proportion in the total population and *IP*_*o*_ = incidence proportion in the unexposed (IA/RA group), and ARR = absolute risk reduction = *IP*_*t*_—incidence proportion in exposed (SA).

Finally, we demonstrated the clinical utility of NAT2 acetylator status and SNPs over clinical variables using ROC analysis and compared the AUCs for prediction models based on clinical factors (“clinical”) and “clinical +genetic” risk factors using DeLong's test [59]. To estimate the additional contribution of genetic factors in a clinical setting, we used all known clinical factors relevant (not invariant) in our cohort, which were age, gender and self-reported ethnicity. The performance of each prediction model (overall sensitivity and specificity) was calculated using AUC of the ROC curves as well as Youden's index (or Youden’s *J* statistic), where *J* = sensitivity + specificity -1. The maximum value of *J* along all points on the ROC curve represents the optimum sensitivity and specificity [60]. All analyses were conducted in PLINK v1.9 [61] or R version 3.3.3 [62].

## Supporting information

S1 Fig Patient recruitment flowchart. Flowchart showing numbers of patients screened, excluded and enrolled for INH-DILI cases and controls.
DILI: drug-induced liver injury, INH: isoniazid, LFT: liver function tests, TB: tuberculosis, TBCU: Tuberculosis control unit, tx: treatment, *: Still attending TBCU for treatment.
(PDF)

S2 Fig Principal components analysis plot. Plot of the first 2 principal components. PC1 separates the Indians from the other ethnicities while PC2 separates the Chinese from the Malays.
(JPG)

S3 Fig Scree plot. Plot of eigenvalues for the first 20 principal components. The first 2 PCs explained more variance than the rest of the PCs.
(JPG)

S4 Fig Cluster plots for associated *NAT2* SNPs. Cluster plots for (a) rs1041983 and (b) rs1495741 shows clear clustering of genotypes.
(JPG)

S5 Fig Predictive value of NAT2 acetylator status and SNPs. Predictive values were evaluated using receiver operating characteristic (ROC) curves and expressed as area-under-curve (AUC), which is a summary measure of the sensitivity and specificity. The clinical model (clin) consists of age, gender and self-reported ethnicity. DOM: dominant, REC: recessive, SA: slow acetylators.
(JPG)

S1 Table Selection of candidate SNPs. (DOCX)

S2 Table Pharmacogenomic association of candidate variants in the Singaporean population. (DOCX)

S3 Table Association results for candidate SNPs with INH-DILI severity. (DOCX)

S4 Table Allelic and genotypic distributions of *NAT2* associated variants. (DOCX)

S5 Table Association of NAT2 acetylator status and SNPs within ethnic groups. (DOCX)

S6 Table Clinical validity of NAT2 acetylator status and 2 significant SNPs by prevalence. (DOCX)

S7 Table Correlation between *NAT2* SNPs and acetylator status. (DOCX)
