# Supplementary figures and images for: Association and clinical utility of NAT2 in the prediction of isoniazid-induced liver injury in Singaporean patients
Source: PLoS One. 2017 Oct 16;12(10):e0186200. doi: 10.1371/journal.pone.0186200 (PMC5642896; doi:10.1371/journal.pone.0186200)

## CASES

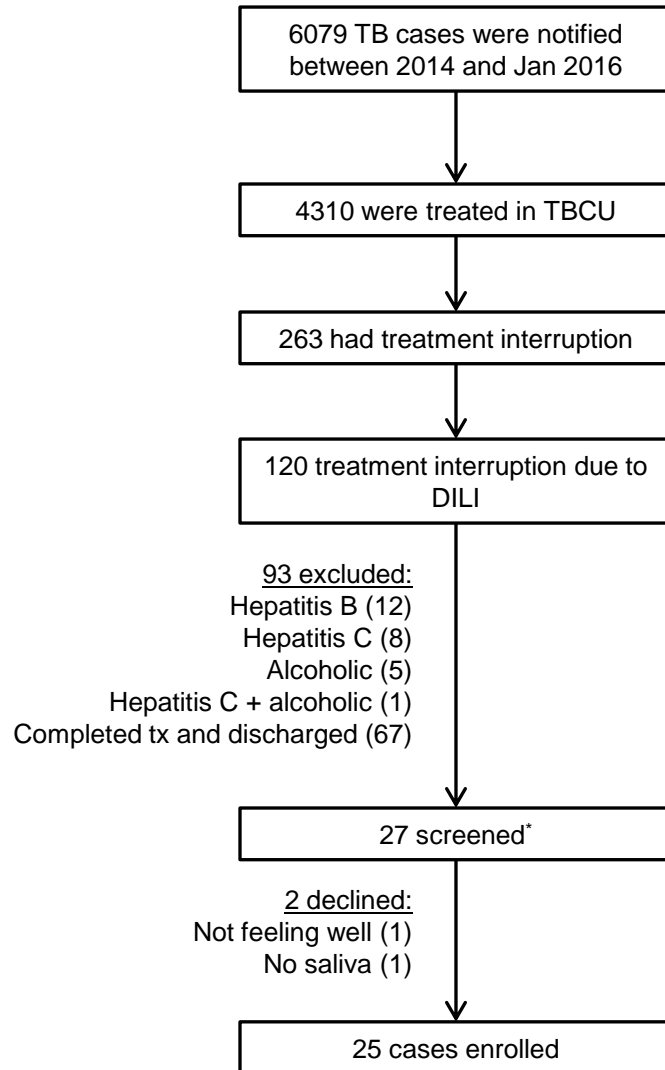

## CONTROLS

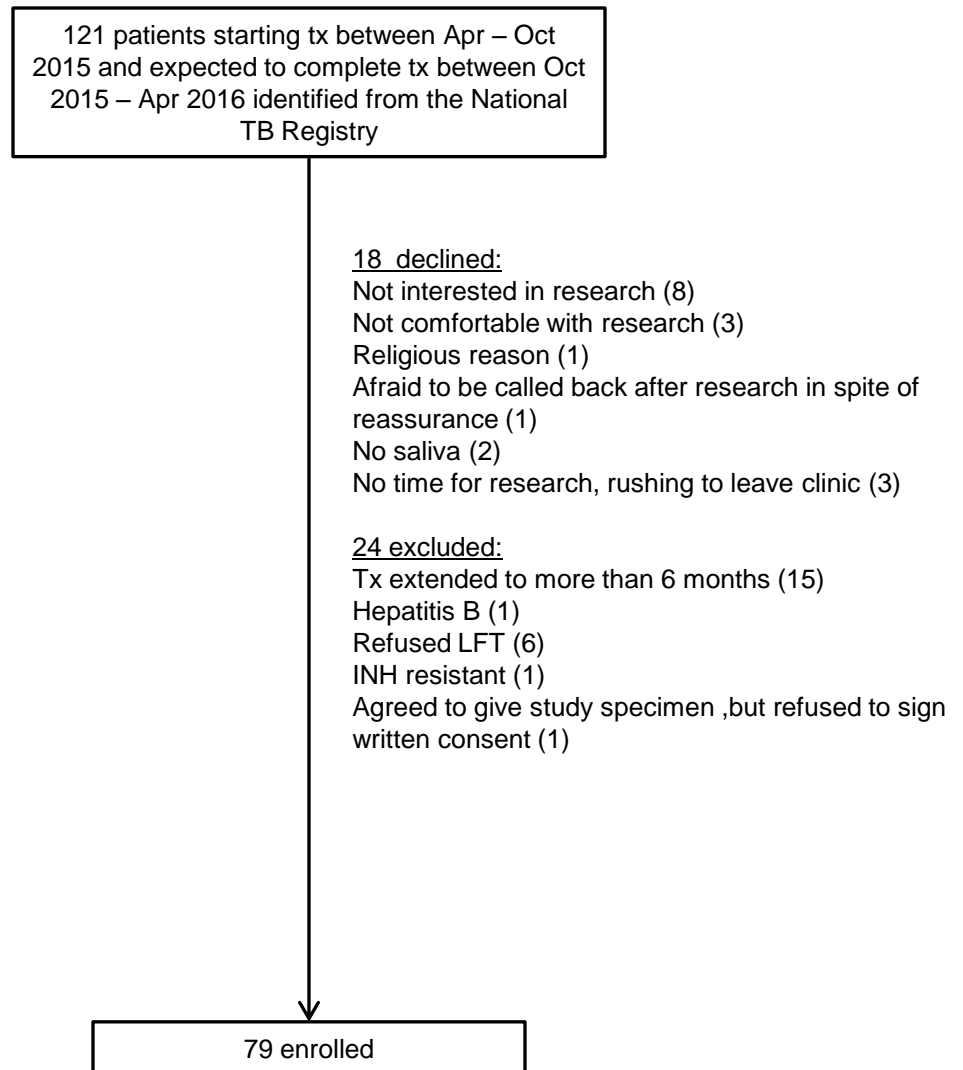

Supplement: S1 Fig — Flowchart showing numbers of patients screened, excluded and enrolled for INH-DILI cases and controls. DILI: drug-induced liver injury, INH: isoniazid, LFT: liver function tests, TB: tuberculosis, TBCU: Tuberculosis control unit, tx: treatment, *: Still attending TBCU for treatment. (PDF) [file pone.0186200.s001.pdf]

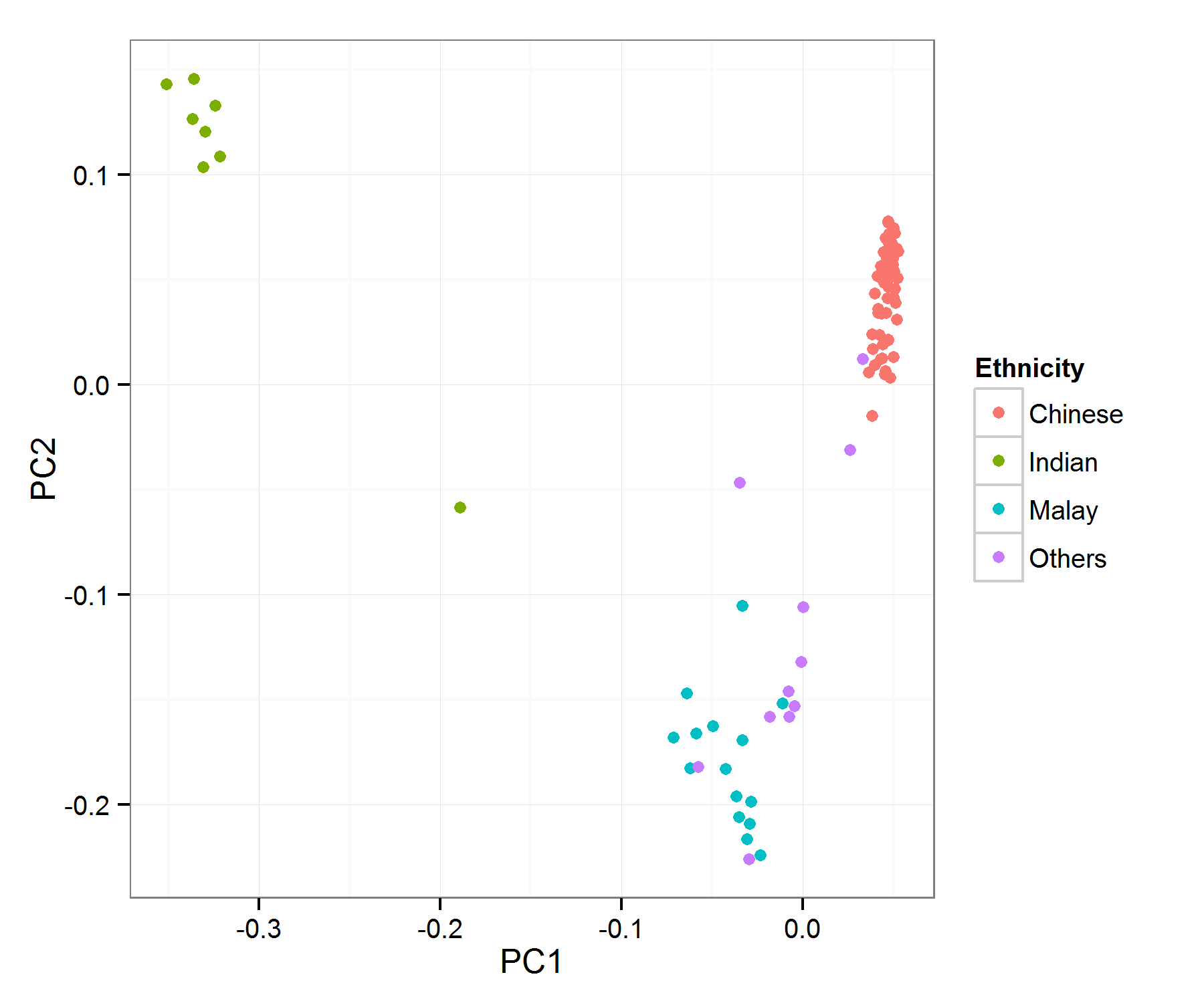

Supplement: S2 Fig — Plot of the first 2 principal components. PC1 separates the Indians from the other ethnicities while PC2 separates the Chinese from the Malays. (JPG) [file pone.0186200.s002.jpg]

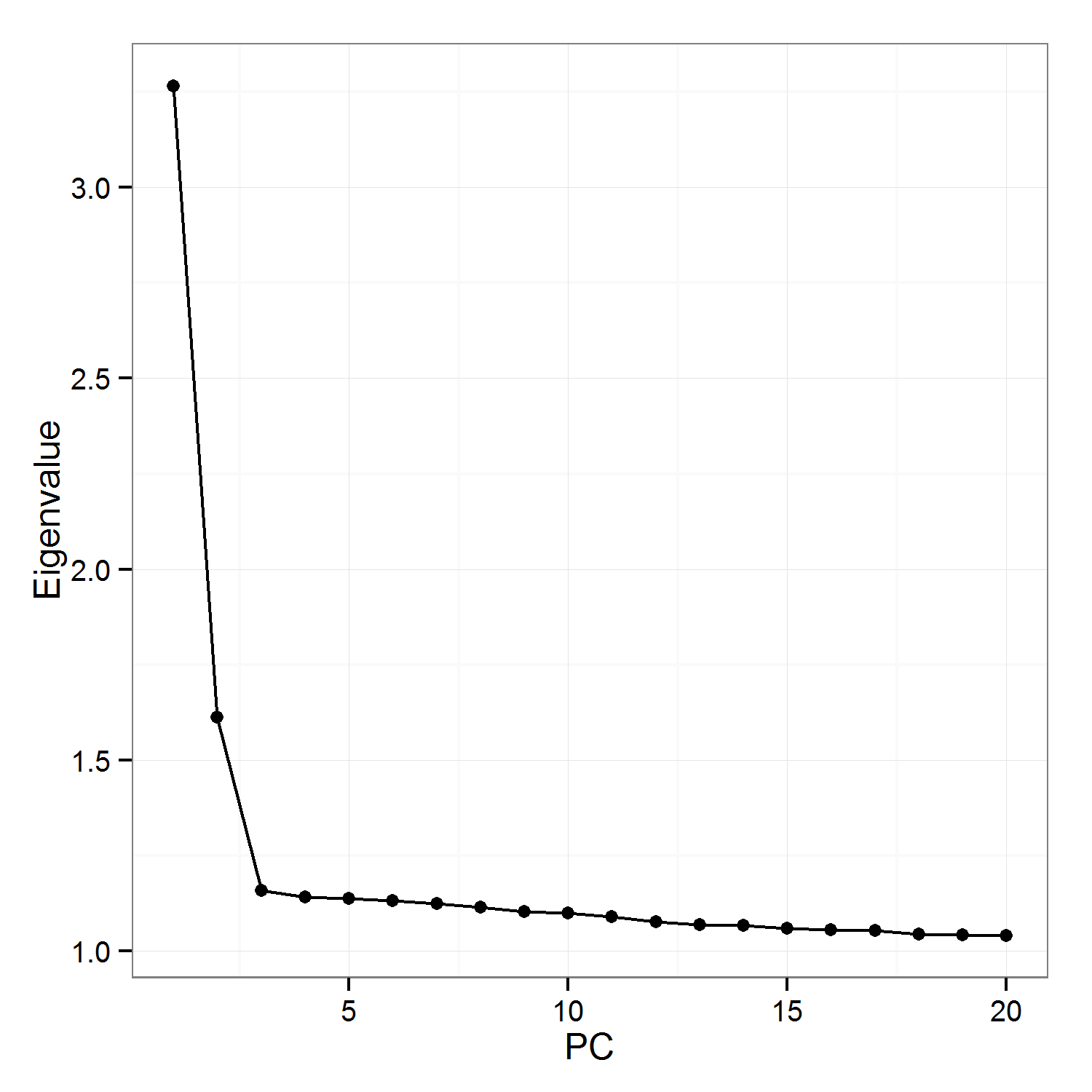

Supplement: S3 Fig — Plot of eigenvalues for the first 20 principal components. The first 2 PCs explained more variance than the rest of the PCs. (JPG) [file pone.0186200.s003.jpg]

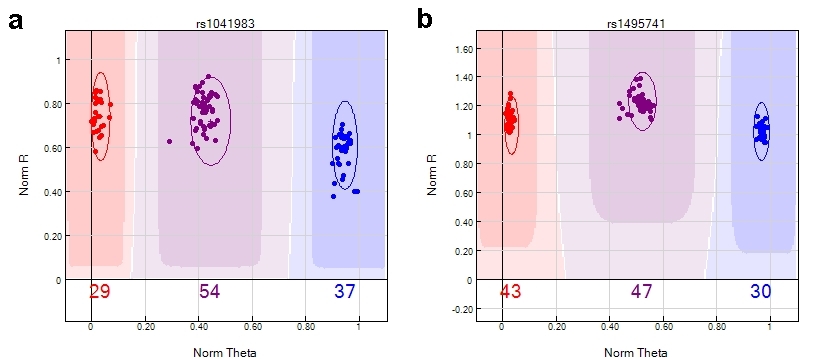

Supplement: S4 Fig — Cluster plots for (a) rs1041983 and (b) rs1495741 shows clear clustering of genotypes. (JPG) [file pone.0186200.s004.jpg]

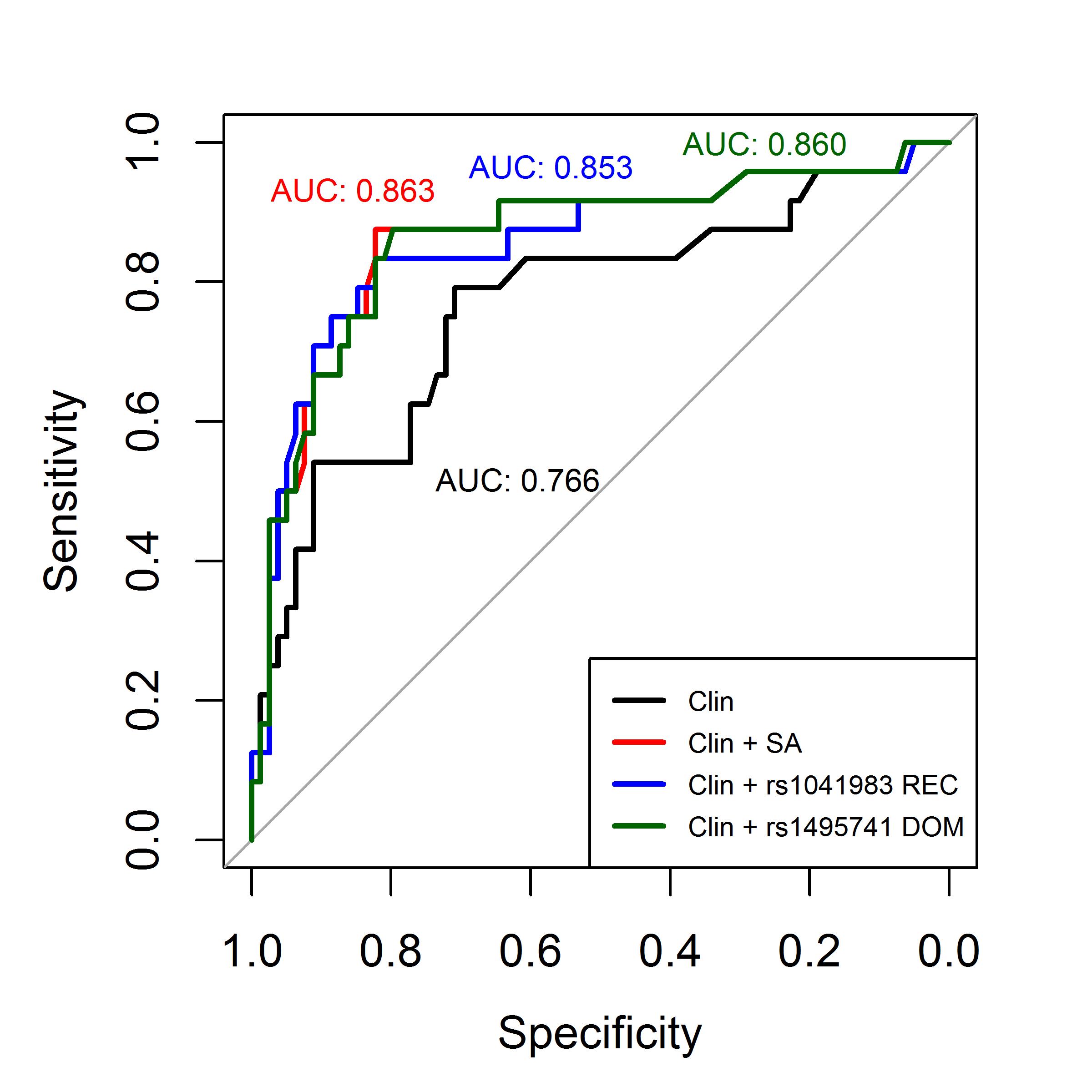

Supplement: S5 Fig — Predictive values were evaluated using receiver operating characteristic (ROC) curves and expressed as area-under-curve (AUC), which is a summary measure of the sensitivity and specificity. The clinical model (clin) consists of age, gender and self-reported ethnicity. DOM: dominant, REC: recessive, SA: slow acetylators. (JPG) [file pone.0186200.s005.jpg]
