# Supplementary material for: Association and clinical utility of NAT2 in the prediction of isoniazid-induced liver injury in Singaporean patients
Source: PLoS One. 2017 Oct 16;12(10):e0186200. doi: 10.1371/journal.pone.0186200 (PMC5642896; doi:10.1371/journal.pone.0186200)
Supplement: S1 Table — (DOCX) [file pone.0186200.s006.docx]

Table S1 Selection of candidate SNPs

| Gene | SNP | Justification/  Reference (PMID) | MAF | | | In panel | Selected |
| --- | --- | --- | --- | --- | --- | --- | --- |
|  |  |  | Chinese | Malays | Indians |  |  |
| *NAT2* | rs1041983 | 1 of 7 common coding SNPs for inferring acetylator status (numerous significant studies^†^). | 0.479 | 0.472 | 0.464 | Y | Y |
|  | rs1799929 |  | 0.042 | 0.079 | 0.271 | Y | Y |
|  | rs1799930 |  | 0.286 | 0.343 | 0.434 | Y | Y |
|  | rs1799931 |  | 0.182 | 0.157 | 0.030 | Y | Y |
|  | rs1801279 |  | 0 | 0 | 0 | N | N |
|  | rs1801280 |  | 0.046 | 0.080 | 0.314 | Y | Y |
|  | rs1208 |  | 0.045 | 0.092 | 0.340 | N | N |
|  | rs4646244 | 19891553 | 0.267^*^ |  | 0.402^*^ | N | N |
|  | rs1495741 | 23407048 | 0.474 | 0.382 | 0.211 | Y | Y |
| *CYP2E1* | rs2031920 | 27062377 | 0.242 | 0.152 | 0.018 | Y | Y |
|  | rs3813867 |  | 0.200^*^ |  | 0.005^*^ | Y | Y |
|  | rs6413432 | 27062377 (NS) | 0.257^*^ |  | 0.206^*^ | N | N |
| *GSTM1* | Null allele | 26046920, 23377313, 23232001 |  |  |  | N | N |
| *GSTT1* | Null allele | 26046920, 23377313, 23232001 (NS) |  |  |  | N | N |
| *GSTP1* | rs1695 | 25798582, 27281183 | 0.148 | 0.218 | 0.263 | Y | Y |
| *CES1* | rs1968753 | 22943824 | 0.367^*^ |  | 0.485^*^ | Y | Y |
|  | rs8192950 |  | 0.200^*^ |  | 0.294^*^ | N | N |
| *ABCB1* | rs1045642 | 22162992 | 0.411 | 0.410 | 0.590 | Y | Y |
| *BACH1* | rs2070401 | 22341855 | 0.250 | 0.101 | 0.006 | Y | Y |
| *CYP2B6* | rs3745274 | 22162992, 25271170 | 0.221 | 0.376 | 0.378 | N | N |
| *HLA-DQB1* | *5 | 25250564 |  |  |  | N | N |
| *MAFK* | rs4720833 | 22341855 | 0.406 | 0.382 | 0.524 | Y | Y |
| *NOS2A* | rs11080344 | 22341855 | 0.479 | 0.506 | 0.349 | Y | Y |
| *SLCO1B1* | rs72559746 | 24491431, 25498879 |  |  |  | N | N |
|  | rs2291075 |  | 0.514^*^ |  | 0.176^*^ | N | N |
|  | rs142101690 |  |  |  |  | N | N |
|  | rs4149014 |  | 0.406 | 0.393 | 0.060 | Y | Y |
| *SOD2* | rs4880 | 17400324 | 0.104 | 0.242 | 0.530 | Y | Y |
| *STAT3* | rs1053023 | 25789467 | 0.357^*^ |  | 0.324^*^ | N | N |
|  | rs1053005 |  | 0.357^*^ |  | 0.324^*^ | Y | Y |
|  | rs1053004 |  | 0.395^*^ |  | 0.554^*^ | Y | Y |
| *TNF* | rs1800629 | 22151084 | 0.130 |  | 0.093^*^ | Y | Y |
| *UGT1A1* | rs35350960 | 22230213 | 0.011 | 0.040 | 0 | N | N |
|  | rs8175347 |  |  |  |  | N | N |
| *XPO1* | rs11125883 | 22341855 | 0.410^*^ |  | 0.196^*^ | Y | Y |

Only SNPs which were significantly associated with INH-DILI in at least 1 study are listed. SNPs were selected if the MAF was >0.05 in at least 1 ethnic group and present in the panel. MAF was extracted from the Singapore Genome Variation Project (40) where available.

^†^all SNPs except rs1208 were also individually significant in at least 1 study (PMIDs 11595069, 21856096, 23875638, 24491431, 19891553, 22506592, 25017831, 26911349, 20392357, 23407048), ^*^From 1000 genomes (CHS and STU).

Y: Yes, N: No, NS: not significant
