# Supplementary material for: Association and clinical utility of NAT2 in the prediction of isoniazid-induced liver injury in Singaporean patients
Source: PLoS One. 2017 Oct 16;12(10):e0186200. doi: 10.1371/journal.pone.0186200 (PMC5642896; doi:10.1371/journal.pone.0186200)
Supplement: S2 Table — (DOCX) [file pone.0186200.s007.docx]

Table S2 Pharmacogenomic association of candidate variants in the Singaporean population

| Gene | SNP | Maj/ min | MAF | | Additive | | | Dominant | | | Recessive | | |
| --- | --- | --- | --- | --- | --- | --- | --- | --- | --- | --- | --- | --- | --- |
|  |  |  | Cases | Ctrl | OR (95%CI) | P | Adj P | OR (95%CI) | P | Adj P | OR (95%CI) | P | Adj P |
| *ABCB1* | rs1045642 | G/A | 0.312 | 0.335 | 0.97  (0.44 - 2.13) | 0.945 | 1 | 0.78  (0.28 - 2.11) | 0.618 | 1 | 1.79  (0.36 - 9.01) | 0.479 | 1 |
| *BACH1* | rs2070401 | A/G | 0.146 | 0.212 | 0.69  (0.26 - 1.87) | 0.469 | 1 | 0.56  (0.18 - 1.73) | 0.314 | 0.999 | 1.75  (0.15 - 20.10) | 0.653 | 1 |
| *CES1* | rs1968753 | A/G | 0.396 | 0.408 | 0.83  (0.42 - 1.64) | 0.591 | 1 | 0.86  (0.31 - 2.41) | 0.781 | 1 | 0.65  (0.18 - 2.37) | 0.514 | 1 |
| *CYP2E1* | rs2031920 | G/A | 0.174 | 0.201 | 0.69  (0.26 - 1.81) | 0.453 | 1 | 0.79  (0.27 - 2.36) | 0.677 | 1 | NA^*^ | | |
| *CYP2E1* | rs3813867 | C/G | 0.174 | 0.192 | 0.72  (0.27 - 1.94) | 0.517 | 1 | 0.79  (0.27 - 2.32) | 0.664 | 1 | NA^*^ | | |
| *GSTP1* | rs1695 | A/G | 0.167 | 0.24 | 0.57  (0.20 - 1.60) | 0.285 | 0.998 | 0.43  (0.14 - 1.28) | 0.128 | 0.963 | 8.40  (0.46 - 151.70) | 0.150 | 0.978 |
| *MAFK* | rs4720833 | G/A | 0.333 | 0.354 | 1.02  (0.45 - 2.32) | 0.970 | 1 | 1.07  (0.37 - 3.10) | 0.903 | 1 | 0.90  (0.15 - 5.36) | 0.903 | 1 |
| *NAT2* | rs1041983 | G/A | 0.792 | 0.367 | **6.34**  **(2.54 - 15.82)** | **7.667 x 10^-5^** | **0.003** | 5.72  (1.18 - 27.69) | 0.030 | 0.597 | **13.86**  **(4.30 - 44.70)** | **1.078 x 10^-5^** | **4.754 x 10^-4^** |
| *NAT2* | rs1495741 | A/G | 0.167 | 0.551 | **0.21**  **(0.09 - 0.52)** | **6.267 x 10^-4^** | **0.024** | **0.10**  **(0.03 - 0.33)** | **1.084 x 10^-4^** | **0.004** | 0.25  (0.05 - 1.21) | 0.085 | 0.901 |
| *NAT2* | rs1799929 | G/A | 0.042 | 0.076 | 0.36  (0.07 - 1.95) | 0.235 | 0.995 | 0.36  (0.07 - 1.95) | 0.235 | 0.995 | NA^*^ | | |
| *NAT2* | rs1799930 | G/A | 0.5 | 0.222 | 2.85  (1.32 - 6.16) | 0.008 | 0.226 | 2.16  (0.74 - 6.28) | 0.159 | 0.980 | 12.00  (2.49 - 57.84) | 0.002 | 0.069 |
| *NAT2* | rs1799931 | G/A | 0.292 | 0.139 | 2.85  (1.20 - 6.77) | 0.017 | 0.420 | 3.10  (1.05 - 9.15) | 0.040 | 0.691 | 7.33  (0.96 - 56.24) | 0.055 | 0.790 |
| *NAT2* | rs1801280 | A/G | 0.042 | 0.095 | 0.30  (0.05 - 1.62) | 0.161 | 0.978 | 0.30  (0.05 - 1.68) | 0.169 | 0.982 | NA^*^ | | |
| *NOS2A* | rs11080344 | G/A | 0.438 | 0.437 | 1.05  (0.51 - 2.14) | 0.901 | 1 | 0.64  (0.23 - 1.80) | 0.396 | 1 | 2.15  (0.64 - 7.21) | 0.213 | 0.994 |
| *SLCO1B1* | rs4149014 | A/C | 0.438 | 0.335 | 1.21  (0.56 - 2.60) | 0.624 | 1 | 0.91  (0.30 - 2.76) | 0.861 | 1 | 2.14  (0.56 - 8.11) | 0.264 | 0.997 |
| *SOD2* | rs4880 | A/G | 0.229 | 0.215 | 1.05  (0.41 - 2.71) | 0.920 | 1 | 1.02  (0.35 - 2.92) | 0.975 | 1 | 1.36  (0.08 - 22.35) | 0.827 | 1 |
| *STAT3* | rs1053004 | A/G | 0.458 | 0.437 | 0.75  (0.35 - 1.60) | 0.453 | 1 | 0.77  (0.25 - 2.38) | 0.646 | 1 | 0.61  (0.16 - 2.27) | 0.457 | 1 |
| *STAT3* | rs1053005 | A/G | 0.417 | 0.38 | 0.94  (0.44 - 2.02) | 0.882 | 1 | 0.94  (0.32 - 2.78) | 0.914 | 1 | 0.91  (0.23 - 3.67) | 0.896 | 1 |
| *TNF* | rs1800629 | G/A | 0.021 | 0.101 | 0.17  (0.02 - 1.43) | 0.103 | 0.934 | 0.17  (0.02 - 1.43) | 0.103 | 0.934 | NA^*^ | | |
| *XPO1* | rs11125883 | A/C | 0.354 | 0.468 | 0.63  (0.31 - 1.29) | 0.205 | 0.992 | 0.53  (0.19 - 1.49) | 0.233 | 0.996 | 0.54  (0.14 - 2.15) | 0.384 | 1 |

This table shows the association results from logistic regression with gender, PC1 and PC2 as covariates. P values were adjusted for 55 multiple correlated tests using the p_ACT procedure ^47^. Significant SNPs (Adj P <0.05) are bolded.

^*^Logistic regression could not be performed due to absence of patients with homozygous variant in either cases or controls.

Adj P: adjusted P value, CI: confidence interval, Ctrl: controls, MAF: minor allele frequency, Maj: major allele, Min: minor allele, OR: odds ratio
