# Supplementary material for: Association and clinical utility of NAT2 in the prediction of isoniazid-induced liver injury in Singaporean patients
Source: PLoS One. 2017 Oct 16;12(10):e0186200. doi: 10.1371/journal.pone.0186200 (PMC5642896; doi:10.1371/journal.pone.0186200)
Supplement: S3 Table — (DOCX) [file pone.0186200.s008.docx]

Table S3 Association results for candidate SNPs with INH-DILI severity

| Gene | SNP | Maj/ min | MAF | | Additive | | | Dominant | | | Recessive | | |
| --- | --- | --- | --- | --- | --- | --- | --- | --- | --- | --- | --- | --- | --- |
|  |  |  | Cases | Ctrl | OR (95%CI) | P | Adj P | OR (95%CI) | P | Adj P | OR (95%CI) | P | Adj P |
| *ABCB1* | rs1045642 | G/A | 0.312 | 0.335 | 0.86  (0.39 - 1.83) | 0.700 | 1 | 0.67  (0.25 - 1.80) | 0.428 | 1 | 1.47  (0.27 - 6.44) | 0.626 | 1 |
| *BACH1* | rs2070401 | A/G | 0.146 | 0.212 | 0.83  (0.30 - 2.13) | 0.712 | 1 | 0.71  (0.22 - 2.09) | 0.547 | 1 | 2.00  (0.09 - 19.58) | 0.583 | 1 |
| *CES1* | rs1968753 | A/G | 0.396 | 0.408 | 0.96  (0.49 - 1.84) | 0.900 | 1 | 1.09  (0.41 - 3.01) | 0.866 | 1 | 0.75  (0.18 - 2.53) | 0.655 | 1 |
| *CYP2E1* | rs2031920 | G/A | 0.174 | 0.201 | 0.73  (0.26 - 1.83) | 0.525 | 1 | 0.84  (0.28 - 2.39) | 0.747 | 1 | NA^*^ | | |
| *CYP2E1* | rs3813867 | C/G | 0.174 | 0.192 | 0.77  (0.27 – 2.00) | 0.603 | 1 | 0.83  (0.27 - 2.37) | 0.737 | 1 | NA^*^ | | |
| *GSTP1* | rs1695 | A/G | 0.167 | 0.240 | 0.64  (0.22 - 1.71) | 0.392 | 1 | 0.49  (0.16 - 1.38) | 0.192 | 0.987 | 11.59  (0.42 - 241.13) | 0.095 | 0.919 |
| *MAFK* | rs4720833 | G/A | 0.333 | 0.354 | 1.02  (0.44 - 2.32) | 0.959 | 1 | 1.06  (0.38 - 3.06) | 0.909 | 1 | 0.92  (0.11 - 5.05) | 0.932 | 1 |
| *NAT2* | rs1041983 | G/A | 0.792 | 0.367 | **6.16**  **(2.65 - 16.19)** | **7.426 x 10^-5^** | **0.003** | 4.99  (1.22 - 34.15) | 0.047 | 0.734 | **12.64**  **(4.21 - 41.28)** | **1.104 x 10^-5^** | **4.681 x 10^-4^** |
| *NAT2* | rs1495741 | A/G | 0.167 | 0.551 | **0.21**  **(0.08 - 0.5)** | **7.735 x 10^-4^** | **0.029** | **0.11**  **(0.03 - 0.32)** | **1.410 x 10^-4^** | **0.006** | 0.29  (0.04 - 1.24) | 0.133 | 0.960 |
| *NAT2* | rs1799929 | G/A | 0.042 | 0.076 | 0.35 (0.05 - 1.57) | 0.217 | 0.993 | 0.35  (0.05 - 1.57) | 0.217 | 0.993 | NA^*^ | | |
| *NAT2* | rs1799930 | G/A | 0.500 | 0.222 | 2.52 (1.18 - 5.47) | 0.017 | 0.416 | 1.87  (0.63 - 5.68) | 0.259 | 0.996 | 7.36  (1.90 - 29.38) | 0.004 | 0.123 |
| *NAT2* | rs1799931 | G/A | 0.292 | 0.139 | 2.88 (1.28 - 6.66) | 0.011 | 0.304 | 2.90  (1.04 - 8.38) | 0.044 | 0.718 | 8.89  (1.34 - 60.88) | 0.02 | 0.467 |
| *NAT2* | rs1801280 | A/G | 0.042 | 0.095 | 0.300  (0.04 - 1.34) | 0.156 | 0.971 | 0.30  (0.04 - 1.38) | 0.162 | 0.975 | NA^*^ | | |
| *NOS2A* | rs11080344 | G/A | 0.438 | 0.437 | 1.13  (0.55 - 2.29) | 0.738 | 1 | 0.68  (0.25 - 1.92) | 0.462 | 1 | 2.47  (0.73 - 7.91) | 0.130 | 0.961 |
| *SLCO1B1* | rs4149014 | A/C | 0.438 | 0.335 | 1.35  (0.62 - 2.92) | 0.443 | 1 | 0.97  (0.34 - 2.95) | 0.960 | 1 | 2.85  (0.71 - 10.78) | 0.126 | 0.959 |
| *SOD2* | rs4880 | A/G | 0.229 | 0.215 | 1.12  (0.43 - 2.85) | 0.805 | 1 | 1.12  (0.39 - 3.13) | 0.824 | 1 | 1.22  (0.05 - 24.39) | 0.890 | 1 |
| *STAT3* | rs1053004 | A/G | 0.458 | 0.437 | 0.76  (0.36 - 1.57) | 0.462 | 1 | 0.89  (0.30 - 2.78) | 0.840 | 1 | 0.51  (0.13 - 1.78) | 0.312 | 0.998 |
| *STAT3* | rs1053005 | A/G | 0.417 | 0.38 | 0.93  (0.44 - 1.92) | 0.837 | 1 | 1.06  (0.37 - 3.13) | 0.915 | 1 | 0.70  (0.16 - 2.61) | 0.615 | 1 |
| *TNFα* | rs1800629 | G/A | 0.021 | 0.101 | 0.14  (0.01 - 0.80) | 0.071 | 0.854 | 0.14  (0.01 - 0.80) | 0.071 | 0.854 | NA^*^ | | |
| *XPO1* | rs11125883 | A/C | 0.354 | 0.468 | 0.57  (0.27 - 1.12) | 0.113 | 0.943 | 0.48  (0.17 - 1.34) | 0.156 | 0.975 | 0.43  (0.09 - 1.53) | 0.234 | 0.994 |

This table shows the association results from ordinal logistic regression with gender, PC1 and PC2 as covariates and DILI grade as the dependent variable. OR represent the odds of transitioning to a higher grade. 95%CIs were obtained by profiling the likelihood function. P values were adjusted for 55 multiple correlated tests using the p_ACT procedure (47). Significant SNPs (Adj P <0.05) are bolded.

*Ordinal logistic regression could not be performed due to absence of patients with homozygous variant in either cases or controls.

Adj P: adjusted P value, CI: confidence interval, Ctrl: controls, MAF: minor allele frequency, Maj: major allele, Min: minor allele, OR: odds ratio
