# Supplementary material for: Association and clinical utility of NAT2 in the prediction of isoniazid-induced liver injury in Singaporean patients
Source: PLoS One. 2017 Oct 16;12(10):e0186200. doi: 10.1371/journal.pone.0186200 (PMC5642896; doi:10.1371/journal.pone.0186200)
Supplement: S4 Table — (DOCX) [file pone.0186200.s009.docx]

Table S4 Allelic and genotypic distributions of *NAT2* associated variants

| Variant | Genotype/ allele | Acetylator status | Cases  (n = 24) | Controls  (n = 79) | OR (95%CI) | P-value^*^ |
| --- | --- | --- | --- | --- | --- | --- |
| rs1041983 | AA |  | 16 | 9 | 24.74 (4.57 – 260.71) | 4.898 x 10^-6^ |
|  | AG |  | 6 | 40 | 2.23 (0.36 – 24.08) | 0.460 |
|  | AA+AG |  | 22 | 49 | 6.64 (1.46 – 62.33) | 0.005 |
|  | GG |  | 2 | 30 | Ref |  |
|  |  |  |  |  |  |  |
|  | AA |  | 16 | 9 | 14.92 (4.60 – 53.97) | 2.852 x 10^-7^ |
|  | AG+GG |  | 8 | 70 | Ref |  |
|  |  |  |  |  |  |  |
|  | A |  | 38 | 58 | 6.49 (2.91 – 15.73) | 3.367 x 10^-7^ |
|  | G |  | 10 | 100 | Ref |  |
|  |  |  |  |  |  |  |
| rs1495741 | GG |  | 2 | 25 | 0.079 (0.008 – 0.396) | 2.657 x 10^-4^ |
|  | GA |  | 4 | 37 | 0.106 (0.023 – 0.385) | 9.294 x 10^-5^ |
|  | GG/GA |  | 6 | 62 | 0.094 (0.026 – 0.294) | 3.036 x 10^-6^ |
|  | AA |  | 18 | 17 | Ref |  |
|  |  |  |  |  |  |  |
|  | G |  | 8 | 87 | 0.165 (0.062 – 0.386) | 2.238 x 10^-6^ |
|  | A |  | 40 | 71 | Ref |  |
|  |  |  |  |  |  |  |
| Acetylator status | *5/*5 | Slow | 0 | 1 | 12.20 (2.42 – 122.36)^†^ | 3.019 x 10^-4^ |
|  | *5/*6 |  | 1 | 7 |  |  |
|  | *5/*7 |  | 1 | 0 |  |  |
|  | *6/*6 |  | 8 | 3 |  |  |
|  | *6/*7 |  | 5 | 4 |  |  |
|  | *7/*7 |  | 3 | 2 |  |  |
|  | *4/*5 | Intermediate | 0 | 5 | 1.26 (0.17 – 14.92) ^†^ | 1 |
|  | *4/*6 |  | 2 | 18 |  |  |
|  | *4/*7 |  | 2 | 14 |  |  |
|  | *5/*13 |  | 0 | 1 |  |  |
|  | *4/*4 | Rapid | 2 | 24 | Ref |  |
|  |  |  |  |  |  |  |
|  |  | Slow | 18 | 17 | 10.62 (3.40 – 38.10) | 3.306 x 10^-6^ |
|  |  | Intermediate/Rapid | 6 | 62 | Ref |  |

^*^Fisher’s exact test, ^†^Calculated using the totals of slow (18 cases & 17 controls) and intermediate (8 cases & 38 controls) acetylators, respectively.

OR: odds ratio, Ref: reference
