# Supplementary material for: Association and clinical utility of NAT2 in the prediction of isoniazid-induced liver injury in Singaporean patients
Source: PLoS One. 2017 Oct 16;12(10):e0186200. doi: 10.1371/journal.pone.0186200 (PMC5642896; doi:10.1371/journal.pone.0186200)
Supplement: S5 Table — (DOCX) [file pone.0186200.s010.docx]

Table S5 Association of NAT2 acetylator status and SNPs within ethnic groups

|  | | n | | OR (95%CI) | P-value^†^ |
| --- | --- | --- | --- | --- | --- |
|  |  | Cases | Controls |  |  |
| Chinese (n = 69) | | 12 | 57 |  |  |
|  | NAT2 SA | 8 | 9 | 10.76 (2.71 – 50.71) | 1.18 x 10^-3^ |
|  | rs1041983 AA | 7 | 6 | 14.79 (3.36 – 82.14) | 7.06 x 10^-4^ |
|  | rs1495741 GA/GG | 4 | 48 | 0.09 (0.019 – 0.369) | 1.18 x 10^-3^ |
|  | |  |  |  |  |
| Malays (n = 15) | | 5 | 10 |  |  |
|  | NAT2 SA | 3 | 3 | 2.48 (0.22 – 31.97) | 0.458 |
|  | rs1041983 AA | 3 | 2 | 4.80 (0.44 – 69.04) | 0.208 |
|  | rs1495741 GA/GG | 2 | 7 | 0.403 (0.031 – 4.54) | 0.458 |
|  | |  |  |  |  |
| Indians (n = 8) | | 1 | 7 |  |  |
|  | NAT2 SA | 1 | 4 | 2.33^*^ (0.02 – Inf)^‡^ | 1^‡^ |
|  | rs1041983 AA | 1 | 0 | 45^*^ (0.18 – Inf) ^‡^ | 0.125^‡^ |
|  | rs1495741 GA/GG | 0 | 4 | 0 (0 – 39.00) ^‡^ | 1^‡^ |

^†^From logistic regression with gender as covariate, ^*^Estimated by adding 0.5 to each cell. ^‡^Estimated from Fisher’s exact test.

CI: confidence interval, Inf: infinity, OR: odds ratio, SA: slow acetylators
