# Supplementary material for: Association and clinical utility of NAT2 in the prediction of isoniazid-induced liver injury in Singaporean patients
Source: PLoS One. 2017 Oct 16;12(10):e0186200. doi: 10.1371/journal.pone.0186200 (PMC5642896; doi:10.1371/journal.pone.0186200)
Supplement: S6 Table — (DOCX) [file pone.0186200.s011.docx]

Table S6 Clinical validity of NAT2 acetylator status and 2 significant SNPs by prevalence

| Prevalence | | 0.05 | 0.1 | 0.15 | 0.2 |
| --- | --- | --- | --- | --- | --- |
| NAT2 SA | | | | | |
|  | Sensitivity | 75% | | | |
|  | Specificity | 78% | | | |
|  | PPV | 16% | 28% | 38% | 47% |
|  | NPV | 98% | 97% | 95% | 93% |
| rs1041983 REC | | | | | |
|  | Sensitivity | 66.7% | | | |
|  | Specificity | 89% | | | |
|  | PPV | 24% | 39% | 51% | 59% |
|  | NPV | 98% | 96% | 94% | 91% |
| rs1495741 DOM | | | | | |
|  | Sensitivity | 75% | | | |
|  | Specificity | 78% | | | |
|  | PPV | 16% | 28% | 38% | 47% |
|  | NPV | 98% | 97% | 95% | 93% |

The risk genotypes for all variants were used for the calculation of clinical validity measures. For rs1495741, the homozygous AA genotype is the risk genotype since GA/GG is protective.

DOM: dominant, NPV: negative predictive value, PPV: positive predictive value, REC: recessive, SA: slow acetylators
