# Supplementary material for: Association and clinical utility of NAT2 in the prediction of isoniazid-induced liver injury in Singaporean patients
Source: PLoS One. 2017 Oct 16;12(10):e0186200. doi: 10.1371/journal.pone.0186200 (PMC5642896; doi:10.1371/journal.pone.0186200)
Supplement: S7 Table — (DOCX) [file pone.0186200.s012.docx]

Table S7 Correlation between *NAT2* SNPs and acetylator status

| SNP | | Acetylator status | | | Concordance (%) |
| --- | --- | --- | --- | --- | --- |
|  |  | SA | IA | RA |  |
| rs1041983 | |  |  |  | 90.3 |
|  | AA | 25 | 0 | 0 |  |
|  | AG | 9 | 37 | 0 |  |
|  | GG | 1 | 5 | 26 |  |
| rs1495741 | |  |  |  | 98.1 |
|  | AA | 34 | 1 | 0 |  |
|  | AG | 1 | 39 | 1 |  |
|  | GG | 0 | 2 | 25 |  |

Concordance was calculated as the proportion of patients with AA genotypes and SA status and AG/GG genotypes and IA/RA status for both SNPs.

IA: intermediate acetylator, RA: rapid acetylator, SA: slow acetylator
